# The Inter-Arm Diastolic Blood Pressure Difference Induced by One Arm Ischemia: A New Approach to Assess Vascular Endothelia Function

**DOI:** 10.1371/journal.pone.0084765

**Published:** 2014-01-13

**Authors:** Weitong Hu, Juxiang Li, Hai Su, Jiwei Wang, Jinsong Xu, Yanna Liu, Ming Huang, Xiaoshu Cheng

**Affiliations:** 1 Research Institute of Cardiovascular Diseases and Department of Cardiology, Second Affiliated Hospital of Nanchang University, Nanchang, Jiangxi, People's Republic of China; 2 Clinical Echocardiographic Laboratory, Second Affiliated Hospital of Nanchang University, Nanchang, Jiangxi, People's Republic of China; University of Otago, New Zealand

## Abstract

**Objectives:**

To evaluate whether inter-arm diastolic blood pressure difference (DBPl-r) induced by one arm ischemia correlates with flow-mediated dilatation (FMD).

**Methods:**

Bilateral arm BPs were simultaneously measured with two automatic devices and right brachial artery diameter (D) was measured by ultrasound technique in 108 subjects (56 hypertensives and 52 normotensives). Following baseline diameter (D0) and BP measurement, right brachial artery was occluded for 5 minutes. The diameter was measured at 1, 1.5 and 2 min, and bilateral BPs measured at 3, 4 and 5 min after occlusion release. Their averages were recorded as post-D and post-BP, respectively. The difference between post-D and D0 (ΔD) was calculated as the percentage increase of artery diameter (ΔD/D0). The BP difference between left and right arms was calculated as BPl-r, and the difference of post- BPl-r and baseline BPl-r was recorded as the net change of BPl-r (ΔBPl-r).

**Results:**

At baseline, bilateral SBPs and DBPs were similar. Right arm ischemia induced significant DBP decline only in the right arm (68.8±12.7 vs 72.6±12.0 mmHg, P<0.05), which led to an increase of ΔDBPl-r (4.00±3.75 vs 0.78±4.47 mmHg, P<0.05). A positive correlation was seen between ΔD/D0 and ΔDBPl-r (r = 0.744, p<0.001). Furthermore, the correlation between age and ΔDBPl-r (r = −0.358, P<0.01) was similar to that between age and D/D0 (r = −0.398, P<0.01). Meanwhile, both ΔDBPl-r and ΔD/D0 were significantly lower in hypertensive patients than in normotensive patients.

**Conclusion:**

The inter-arm DBP difference induced by one arm ischemia may be a potential index for clinical evaluation of vascular endothelial function.

## Introduction

Vascular endothelium plays a pivotal role in maintaining normal function of blood vessels because it can synthesize biological active substances that regulate vascular tone [1]. As endothelial dysfunction can result from and/or contribute to several disease processes, including hypertension, hypercholesterolaemia and diabetes, evaluation of vascular endothelial function becomes an important clinical examination.

At present, several noninvasive methods are used to evaluate vascular endothelial function, such as laser Doppler fluximetry for capillaries [2], peripheral artery tonometry for digital arteries [3], [4], and flow-mediated dilatation (FMD) method for muscular arteries, like brachial artery. As the most used method, the principle of FMD is to assess the dilatation of brachial artery using ultrasound technique during post-ischemic reactive hyperemia, when the artery is exposed to increased flow and shear stress[5], [6].

Many studies have demonstrated that exercise can lead to reactive hyperemia and post exercise hypotension like short-term ischemia[7]–[9]. Our previous studies showed that 3-min one-arm exercise induced marked diastolic BP (DBP) decline only in the exercised arm, followed by increase in the inter-arm DBP difference [10]. Furthermore, the inter-arm DBP difference induced by one-arm exercise was age-dependent [11]. However, this action did not induce inter-arm systolic BP difference because the SBP of both arms changed simultaneously during the observation period.

As both acute ischemia and acute exercise can induce reactive hyperemia, we hypothesized that a 5-min ischemia of right arm in FMD test might also cause DBP decline in the ischemic arm and subsequently induces significant DBP difference between the left and right arms (DBPl-r); meanwhile, the extent of DBPl-r might correlate with the dilatation of brachial artery. If this hypothesis is true, the DBPl-r induced by one arm ischemia may be used to assess the vasodilatation capacity. This study is to test the hypothesis.

## Subjects and Methods

The proposal and the consent procedures of this study were approved by the Ethic Committees of the Second Affiliated Hospital of Nanchang University. After explaining the value of examination, all patients provided verbal informed consent for BP measurement of bilateral arms and FMD test as both of them are non-invasive clinical examination, and as the test results were recorded only in medical record. Then a research assistant nurse took their names and telephone numbers for following up.

From July to September of 2012, 108 subjects (57 males and 51 females, mean age 51.6±18.2 y) were enrolled. Of them, 56 were hypertensive patients (30 under antihypertensive therapy) and 52 were normotensive subjects. The characteristics of two groups with hypertension or normotension are shown in Table 1. All subjects had normal heart arrhythmia.

**Table 1:** The characteristics of two groups with either hypertension or normotension.

	Hypertension(56)	Normotension(52)	P value
Male (%)	28(50)	29(56)	0.548
Age (y)	58.9±13.9	50.7±18.3	0.078
BMI (kg/m^2^)	24.2.±7.7	22.9±8.5	0.549
SBP(mm Hg)	138.7±19.6	113.9±12.4	<0.001
DBP(mm Hg)	74.4±12.6	63.6±10.1	<0.001
Diabetes mellitus (%)	13(23.2)	4(7.7)	0.027
Hyperlipidemia (%)	21(37.5)	9(17.3)	0.019
CHD (%)	2(3.6)	3(5.8)	0.587
Other heart disease (%)	3(5.4)	5(9.6)	0.399

CHD: Coronary heart disease.

### Bilateral BP measurement and flow-mediated dilatation test

Examination was performed in an air-conditioned room with a temperature of 22–23°C. Before BP measurement, subjects were asked to empty bladder and take a 10 min rest. The subjects sat with two arms in a comfortable position for imaging right brachial artery and for bilateral BP measurement. Bilateral BPs were simultaneously measured with two automatic BP measurement devices(Omron, HEM-7112). Brachial artery diameter was measured by color Doppler (ALOKA-á7) with a probe of 7.5 MHz. The brachial artery was imaged above the antecubital fossa in the longitudinal plane. A segment with clear anterior and posterior intimal interfaces between the lumen and vessel wall was selected for continuous 2D grayscale imaging. In addition, M mode was recorded for measuring diameter [7].

The diameter of right brachial artery at resting state was measured as the baseline value (D0), and then BPs of both arms were simultaneously measured twice with a 2-min interval and their average was taken as the baseline BP (BP0).

After D0 and BP0 taking, a narrow sphygmomanometric cuff was placed above the antecubital fossa of the right arm. Then arterial occlusion was created by cuff inflation to 30 mmHg above systolic BP for 5 minutes, which was then rapidly deflated. For technical reason, brachial artery diameter and arm BP could not be taken simultaneously. So the diameter of right brachial artery was measured at 1(D1), 1.5 (D1.5) and 2 (D2) min, and bilateral arm BPs were measured at 3 (BP3), 4 (BP4) and 5 (BP5) min after the release of the occlusive cuff, respectively.

### Parameters

#### FMD

The images of individual subjects were analyzed by one investigator. Brachial arterial diameter (D) was measured from the anterior to the posterior interface between the media and adventitia at the onset of R-wave of ECG.

Percentage increase of artery diameter (ΔD/D0): The average of D1, D1.5 and D2 was defined as post-D for assessing the increase of artery diameter ΔD (ΔD =  post-D - D0). Then the percentage increase of artery diameter was calculated as (ΔD/D0) (5).

#### BP

Two BP parameters were calculated in this study.

BPb-p was the BP difference before and after ischemia in the right arm (BPb-p =  baseline BP - post-ischemia BP). Here the post-ischemia BP was the average of BPb-p3, BPb-p4 and BPb-p5.

ΔBPl-r was the difference between post-ischemia BPl-r and baseline BPl-r, which reflects the net change of BPl-r induced by right arm ischemia. Here, the BPl-r was the BP difference between left and right arms (BPl-r  =  left BP- right BP), and the post-ischemia BPl-r was the average of BPl-r3, BPl-r4 and BPl-r5.

The higher baseline BP of two arms was used for individual baseline BP.

### Statistical analysis

Data were processed in Excel 2003 and analyzed with SPSS10.0. Continuous variables were expressed as mean ±SD. The t-test and the analysis of variance (ANOVA) were used for the statistical analysis. Lineal regress analysis was performed for the correlation between ΔD/D0 and BP parameters, as well as the correlations of ΔD/D0 and ΔBPl-r with age or baseline BP levels. A value of less than 0.05 was considered statistically significant.

## Results

The characteristics of the two subject groups with either hypertension or normotension are showed in Table 1. The SBP and DBP levels, as well as the percentages of diabetes and hyperlipidemia were higher in the hypertensive group, while the age, BMI, gender distribution and incident of heart diseases were similar between the two groups (Table 1).

### Bilateral BP

The SBP and DBP of both arms were similar at baseline and the values of either SBPl-r or DBPl-r were very small. As right arm ischemia only induced significant DBP decline in the right arm, the post-DBPl-r significantly increased following right arm ischemia. However, right arm ischemia did not induce significant change in bilateral SBPs or in post-SBPl-r (Table 2).

**Table 2:** The BP of both arms before and after right arm ischemia (n = 108).

	SBP(mmHg)			DBP(mmHg)		
	Left arm	Right arm	SBPl-r	Left arm	Right arm	DBPl-r
Baseline	128.0±21.4	129.3±21.7	−1.34±4.10	72.6±11.9	72.6±12.0	−0.03±2.51
Post-	125.3±20.7	125.9±20.4	−0.56±4.47	72.6±12.1	68.8±12.7* #	3.97±3.61*
BPb-p	2.71±5.93	3.49±6.95	/	0.03±2.51	3.79±4.49#	/
ΔBPl-r	/	/	0.78±4.47	/	/	4.00±3.75*

*compared with baseline p<0.05;

^#^ compared with left arm.

Post-: after right arm ischemia; BPb-p: same-arm BP differences before and after ischemia; ΔBPl-r: BPl-r change following ischemia; (BPl-r: BP difference between left and right arms).

### The correlation between ΔD/D0 and BP parameters

**Figure 1:**
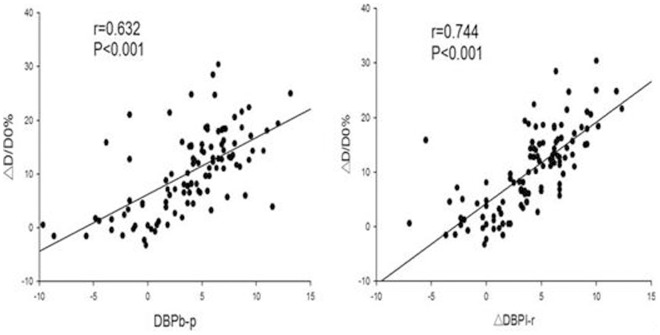
The correlation of ΔD/D0 with DBPb-p and ΔDBPl-r. △D: increase in artery diameter (ΔD =  post-D - D0). ΔD/D0: percentage increase in right brachial artery diameter. DBPb-p: DBP change following right-arm ischemia. ΔDBPl-r: DBPl-r change following ischemia. (BPl-r: DBP difference between left and right arms).

We observed a positive correlation between ΔD/D0 and DBPb-p of right arm (r = 0.632, P<0.0001) and between ΔD/D0 and ΔDBPl-r (r = 0.744, P<0.0001) (Figure 1). We did not observe any correlation between ΔD/D0 and ΔSBPl-r. However, a weak positive correlation was seen between D/D0 and right arm SBPb-p (r = 0.261, P = 0.006) as well as between D/D0 and left arm SBPb-p (r = 0.217, P = 0.024).

**Figure 2:**
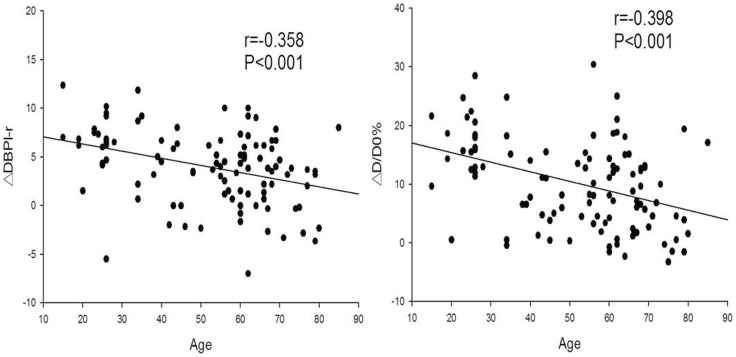
The correlation of age with ΔDBPl-r and ΔD/D0. ΔD/D0: percentage increase in right brachial artery diameter. ΔDBPl-r: DBPl-r change following ischemia. (DBPl-r: The DBP difference between left and right arms).

Like ΔD/D0, ΔDBPl-r was negatively correlated with age with similar coefficients (0.398 and 0.358, both P<0.01) (Figure 2).

**Figure 3:**
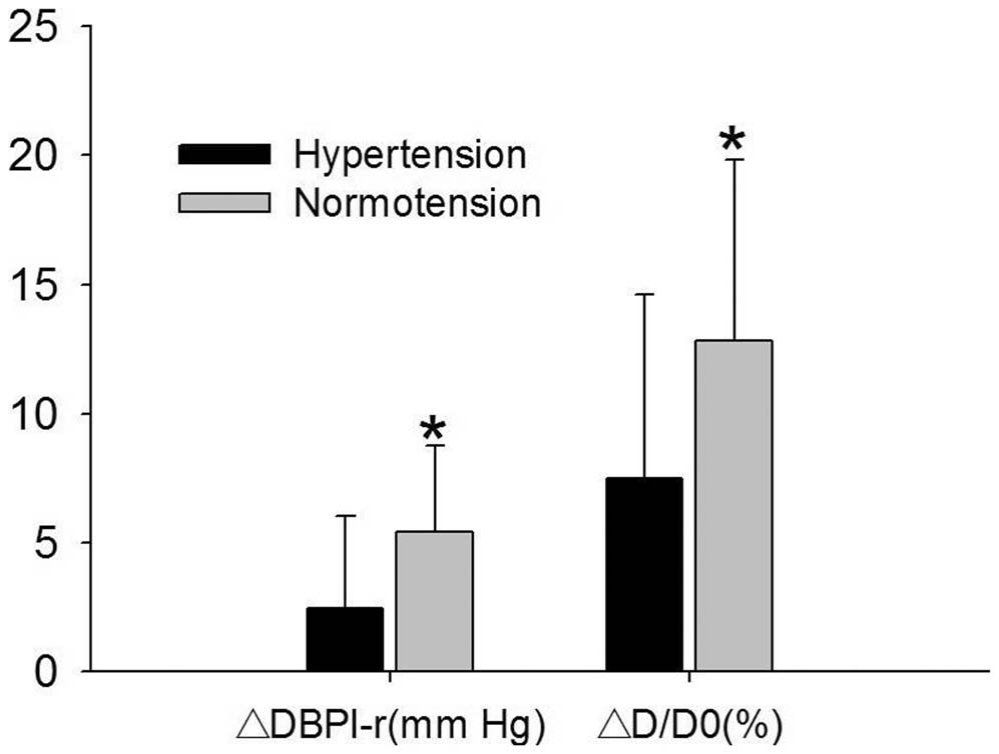
Comparison of ΔD/D0 and ΔDBPl-r between groups with hypertension or normotension. * P<0.001 compared with hypertension group. ΔD/D0: percentage increase in right brachial artery diameter. ΔDBPl-r: DBPl-r change following ischemia. (DBPl-r: DBP difference between left and right arms).

Notably, the ΔDBPl-r was significantly higher in the normotensive group than in the hypertensive group. Similarly, we also observed higher percentage increase in right brachial artery diameter (ΔD/D0) in the normotensive group (Figure 3).

## Discussion

Flow-mediated dilatation (FMD) is a useful method for evaluating vascular endothelial function [12]–[14]. Usually, the maximal increase in artery diameter occurs within 60 to 120 seconds following release of the occlusive cuff [1]. Therefore, in this study the diameter was measured 60, 90 and 120 seconds after cuff release, the average of which was used to calculate ΔD/D0 and to evaluate the dilatation capability of brachial artery.

In the current study, we first demonstrated that 5 min ischemia of right arm induced DBP decline in the right arm, leading to the increase in the DBP difference between baseline and post-ischemia (DBPb-p). At the same time, the post-ischemia inter-arm DBP difference (DBPl-r) increased as well. Because DBPl-r is dependent on the DBP change of both arms, it is a better index than DBPb-p for evaluating vascular endothelial function as the impact of random environmental factors during the observation period is eliminated. Because ΔDBPl-r is calculated as the difference of post-ischemic DBPl-r and baseline DBPl-r, it could reflect the net change of DBPl-r induced by right arm ischemia. Therefore, we focused on ΔDBPl-r rather than DBPl-r in this study.

Secondly, the present study demonstrated that the DBPb-p was positively correlated with the percentage increase in artery diameter (ΔD/D0) in the right arm. This finding demonstrated that ischemia-induced vascular dilatation is the underlying mechanism for the DBP decline in ischemic right arm. Furthermore, a stronger correlation was found between ΔDBPl-r and ΔD/D0. This result further indicates that ΔDBPl-r is more suitable for evaluating the vascular endothelial function compared with DBPb-p.

Thirdly, the present study revealed that the negative coefficient between ΔDBPl-r and age was similar to that between ΔD/D0 and age. Moreover, both DBPl-r and ΔD/D0 were significantly lower in the hypertensive group than in the normotensive group. As age and hypertension are two important risk factors for vascular endothelial dysfunction [1], these results suggest that ΔDBPl-r has similar power to assess vascular endothelial function as ΔD/D0.

Like one arm exercise, one arm ischemia did not induce inter-arm SBP difference, because this procedure induces synchrony SBP change in both arms. However, why one arm exercise or one arm ischemia induces inter-arm diastolic BP difference but not inter-arm systolic BP difference remains unclear. To interpret this phenomenon, we measured the bilateral brachial artery internal diameter (ID), peak systolic flow velocity (PSV) and end diastolic flow velocity (EDV) in 20 young males using Doppler technology before and 5 min after the right-arm ischemia. At baseline, the SBP, DBP, ID, PSV and EDV were similar between two arms. Right arm ischemia only induced slight dilatation of right brachial artery. However, right arm ischemia induced bilateral PSV increase, while the PSV of the right arm was significantly higher than that of the left arm. These results indicate that right arm had larger systolic blood flow volume, which counteracted the vascular resistance decline to maintain the SBP at the similar level of the left arm. As the end diastolic flow velocity of the right brachial artery was similar to that of the left arm, and there was no additional increase in blood flow volume to counteract the lower vascular resistance, the DBP of right arm was lower than that of left arm. However, this finding is only preliminary, and more research is needed to fully understand the mechanism underlying the different changes between SBP and DBP following one arm ischemia.

Although in this study we observed a decrease of about 3 mmHg in the bilateral SBP throughout the experiment, which resulted in a weakly positive correlation between SBPb-p and ΔD/D0 in both arms, we considered this correlation a pseudomorph from SBP decline during the observation period. Although a 10-min rest was taken before BP measurement in our study, this duration may not be enough for a real resting BP. Similar result was reported by Sala et al., in which they found a significant decrease in SBP by 11.6 mm Hg during a 16-min rest on chairs for 55 untreated essential hypertensive patients [15].

### The clinical implication

Compared with FMD, one arm ischemia-induced DBP difference is a simpler and cheaper index as there is no need for special equipment, which may become a way for clinical evaluation of endothelial function, at least as a preliminary screening method.

For technical reason, bilateral arm BPs were measured at 3, 4 and 5 min, but not within 1–2 min after release of occlusive cuff, during which the maximal artery dilatation occurs. It is very possible the correlation between ΔDBPl-r and ΔD/D0 may be higher than that reported in this study if ΔDBPl-r is determined within the 1–2 min duration after release of occlusive cuff.

### Limitation

Because of technical difficulty, the brachial artery diameter and BP could not be simultaneously measured. As many factors can influence vascular endothelia function, for example, diet, time and exercise, repeated test must be performed to measure BP and artery diameter at the same time points after release of occlusive cuff. After careful consideration of the possible impact of different dates and two ischemia procedures in a short period, we used a sequential measurement in this study.

However, simultaneous measurement of BP and artery diameter after release of occlusive cuff may be worthy of future studies.

## Conclusion

As a positive correlation exists between flow-mediated dilatation of brachial artery and inter-arm DBP difference after one arm ischemia, the post-ischemia inter-arm DBP difference may be a potential index for clinical evaluation of vascular endothelial function.
